# Results from the French National Esophageal Atresia register: one-year outcome

**DOI:** 10.1186/s13023-014-0206-5

**Published:** 2014-12-11

**Authors:** Anne Schneider, Sébastien Blanc, Arnaud Bonnard, Naziha Khen-Dunlop, Frédéric Auber, Anne Breton, Guillaume Podevin, Rony Sfeir, Virginie Fouquet, Catherine Jacquier, Jean-Louis Lemelle, Frédéric Lavrand, François Becmeur, Thierry Petit, Marie-Laurence Poli-Merol, Frédéric Elbaz, Thierry Merrot, Jean-Luc Michel, Allal Hossein, Manuel Lopez, Edouard Habonimana, Cécile Pelatan, Pascal De Lagausie, Philippe Buisson, Philine de Vries, Jean Gaudin, Hubert Lardy, Corine Borderon, Joséphine Borgnon, Olivier Jaby, Dominique Weil, Didier Aubert, Stephan Geiss, Jean Breaud, Anis Echaieb, Jane Languepin, Christophe Laplace, Myriam Pouzac, François Lefebvre, Frédéric Gottrand, Laurent Michaud

**Affiliations:** Reference Center for Congenital Esophageal Anomalies, University Hospital Lille, Avenue Eugène Avinée, 59037 Lille, France; University Hospital, Lyon, France; Hôpital Robert Debré, Paris, France; Hôpital Necker Enfants Malades, Paris, France; Hôpital Trousseau, Paris, France; University Hospital, Toulouse, France; University Hospital, Nantes, France; Hospital Kremlin-Bicêtre, Le Kremlin-Bicêtre, France; University Hospital, Grenoble, France; University Hospital, Nancy, France; University Hospital, Bordeaux, France; University Hospital, Strasbourg, France; University Hospital, Caen, France; University Hospital, Reims, France; University Hospital, Rouen, France; Hôpital Nord, Marseille, France; Hôpital Felix Guyon, St Denis, France; University Hospital, Montpellier, France; University Hospital, St Etienne, France; University Hospital, Rennes, France; University Hospital, Le Mans, France; Hôpital La Timone, Marseille, France; University Hospital, Amiens, France; University Hospital, Brest, France; University Hospital, Poitiers, France; University Hospital, Tours, France; University Hospital, Clermont-Ferrand, France; University Hospital, Dijon, France; University Hospital, Créteil, France; University Hospital, Angers, France; University Hospital, Besançon, France; Hôpital Le Parc, Colmar, France; University Hospital, Nice, France; University Hospital, Fort de France, France; University Hospital, Limoges, France; University Hospital, Pointe-à-Pitre, France; Regional Hospital, Orléans, France; Department of Biostatistics, University Hospital, Strasbourg, France

**Keywords:** Esophageal atresia, Population-based registry, One-year outcome, Long-term follow-up

## Abstract

**Background:**

The aim of the present national prospective population-based study was to assess the early morbidity of esophageal atresia (EA).

**Methods:**

All 38 multidisciplinary French centers that care for patients with EA returned a specific questionnaire about the 1-year outcome for each patient. This information was centralized, checked, and entered into a database.

**Results:**

From the total population of 307 EA patients born in 2008 and 2009, data about the 1-year outcome were obtained from 301 (98%) patients, of whom 4% were lost to follow-up and 5% died. Medical complications occurred in 34% of the patients: anastomotic leaks (8%), recurrent tracheoesophageal fistula (4%), and anastomotic stenosis (22%); all of the latter group needed dilation (median, 2 dilations/patient). A new hospitalization was required for 59% of patients (2.5 hospitalizations/patient) for digestive (52%) or respiratory (48%) reasons. Twelve percent of patients required antireflux surgery at a median age of 164 days (range, 33–398 days), and 1% underwent an aortopexy for severe tracheomalacia. The weight/age Z-score was −0.8 (range, −5.5 to 3.7 months) at 12 months. Fifteen percent of patients were undernourished at 12 months of age, whereas 37% presented with respiratory symptoms and 15% had dysphagia at the last follow-up. Significant independent factors associated with medical complications were anastomotic esophageal tension (p = .0009) and presence of a gastrostomy (p = .0002); exclusive oral feeding at discharge was associated with a decreased risk of complications (p = .007).

**Conclusions:**

Digestive and respiratory morbidities remain frequent during the first year of life and are associated with difficult anastomosis and lack of full oral feeding.

**Electronic supplementary material:**

The online version of this article (doi:10.1186/s13023-014-0206-5) contains supplementary material, which is available to authorized users.

## Background

Esophageal atresia (EA) with or without a tracheoesophageal fistula (TEF) is a rare congenital malformation (MIM 189960) [1,2]. In 2012, the EUROCAT Working Group (23 European registries of congenital anomalies) published an overall prevalence of 2.4 per 10 000 births with important regional differences [3]. A prospective population-based register was initiated in 2008 and included all the centers that care for EA patients in France. This register showed that the live-birth prevalence of EA was 1.8 per 10 000 births in our country [4].

The prognosis of EA has benefited from advances in medical care, including neonatal and surgical procedures, and has therefore improved significantly over the past three decades. Its survival rate now exceeds 95% and an increasing number of patients reach adulthood [3,4]. However, retrospective monocentric studies from tertiary centers have reported respiratory and digestive/nutritional problems both in early infancy and at long-term follow-up [5]. Updated information on the outcome of EA from population-based studies is lacking.

In the present study, we report the first-year outcome of all EA patients included in the French register over a 2-year period, as well as factors that were associated with morbidity.

## Methods

A network of all the centers in France that treat EA was created in 2006 within the framework of the French national plan for rare diseases, and this network is coordinated by the National Reference Center for EA located at the Lille University Hospital. A population-based registry of EA was created and began to collect data prospectively on all patients born with EA in France from January 1, 2008. The register was approved by the National Informatics and Privacy Committee (CNIL), and was qualified by the National Committee of Register (InVs, CNR). All data were used anonymously, and the parents were informed about the aims of the register. Two specific questionnaires collected information at birth and at 1 year of age [see Additional file 1]. These questionnaires were validated by a multidisciplinary national committee of experts, including epidemiologists, neonatologists, surgeons, and pediatricians. The questionnaires were completed by the participating centers on a voluntary basis. A clinical research assistant helped to collect the information at each center, when required. A physician and a research assistant checked each questionnaire and double-checked the data entered into the database. When inconsistencies or lack of information were found, the corresponding center was contacted to resolve the issue. The methodology and exhaustivity of the register have been reported elsewhere [4].

The present study concerned the first-year outcome of all EA patients included in the registry who were born between January 1, 2008, and December 31, 2009. EA was classified according to Ladd’s classification [6]. Data collected included general neonatal characteristics of the patient and outcome up to 1 year of age: death, postsurgical direct esophageal complications and chronic complications, new hospitalization and causes, and nutrition status at 6 and 12 months evaluated by the Z-score for weight/age. Undernutrition was defined as a Z-score ≤ −2.0 standard deviation (SD). A medical complication was defined as the occurrence of anastomotic leaks (radiological leaks), recurrent TEF, or anastomotic stenosis needing esophageal dilation. The presence of esophageal anastomotic tension, difficulty in performing anastomosis and timing of primary or delayed primary repair were decided by the surgeon. The variable time to esophageal anastomosis was recorded separately for patients receiving a primary anastomosis (during the neonatal period) and those receiving a delayed primary anastomosis (after a few weeks). Respiratory symptoms were defined as recurrent lung infections or chronic wheezing or recurrent episodes of bronchiolitis, and dysphagia as difficulty in swallowing.

### Statistical analyses

Continuous variables were analyzed using Student’s *t* test or Wilcoxon’s rank-sum test in cases of heteroscedasticity or nonnormality. Categorical variables were analyzed using Fisher’s exact test. The variables with a p-value <0.2 in a univariate analysis and all variables potentially associated with the complication based on clinical knowledge were used in a multivariate logistic regression only if the missing data were less than 30% for the variable. The selection of the variables in the final model was made using backward elimination. The type I error was set at 5% (two-sided) for all statistical tests. R software, version 3.1.0, was used for data analysis.

## Results

From the total population of 307 EA patients born in 2008 and 2009 in France, data about the 1-year outcome were obtained from 301 (98%) patients, of whom 4% (n = 12) were lost to follow-up and 5% died (n = 16). The first-year outcome was available for 275 patients (90%), and their general neonatal characteristics are summarized in Table 1.

**Table 1:** **Neonatal characteristics of the population studied**

**Sex**	**Male 55%**
	**Female 45%**
**Term**	**37 ± 3 weeks**
**Birth weight**	**2591 ± 699 g**
**LADD I [** 6 **]**	**9%**
**LADD III [** 6 **]**	**87%**
**Other EA types [** 6 **]**	**II: 1.0%; IV: 1.5%; V: 1.5%**
**Associated malformation**	**48%**
**Delayed primary anastomosis**	**13%**
**Defect length**	**1.9 ± 1.4 cm**

A total of 16 patients (5%) died during the first year of follow-up, at a median age of 13 days (range, 1–284 days). The causes of death were the consequences of associated malformations or complications of surgery (Table 2).

**Table 2:** **Ages and causes of death**

	**Age at death (days)**	**Term (weeks)**	**Birth weight (g)**	**EA type (LADD)**	**Cause of death**
1	?	39	2300	III	Multivisceral failure (trisomy 18)
2	1	37	2770	III	Multiple malformations
3	1	31	1750	III	Multiple malformations
4	2	34	1760	I	Multiple malformations
5	2	39	1840	III	Cardiorespiratory failure (Fanconi disease)
6	3	36	2100	III	?
7	6	36	1985	III	?
8	6	34	1815	III	Enterocolitis
9	13	28	600	III	?
10	20	35	2380	I	Gastric perforation, sepsis
11	53	30	1295	I	Enterocolitis
12	102	32	1300	III	?
13	151	34	1101	III	Severe sepsis
14	182	38	2190	III	Cardiac failure (sepsis)
15	192	36	2920	I	Mediastinitis
16	284	37	2800	III	Sudden infant death (negative autopsy)

During the first year of follow-up, rehospitalization was required for 59% (160/272) of the patients, with a mean number of hospitalizations of 2.5/patient (±2.0; range, 1.0–13.0 hospitalizations) and a mean hospital stay of 21 days (±37; range, 1–365 days). The main causes of hospitalization were digestive in 52% of patients (e.g., esophageal stenosis, gastroesophageal reflux disease, nutritional evaluation, anorectal malformation surgery, pyloric stenosis, and gastroenteritis…) and respiratory in 48% of patients (e.g., bronchiolitis, respiratory infection, and tracheomalacia…).

A medical complication, as defined previously, occurred in 34% (91/271) of the patients: anastomotic leaks (8%), recurrent TEF (4%), and anastomotic stenosis (22%) needing esophageal dilation in 22% of patients (2.3 dilations/patient; range, 1.0–9.0 dilations; at a mean age of 123 days (range, 20–360 days) for the first dilation). Twelve percent of patients required antireflux surgery at a median age of 164 days (range, 33–398 days), and 1% of patients required an aortopexy for severe tracheomalacia (median age, 122 days; range, 75–238 days).

The percentage of patients receiving a gastrostomy in our 1-year follow-up population was 22%. Most gastrostomies were placed surgically at birth in cases of delayed anastomosis for pure EA or for long defects in type III patients. In some cases, patients with pure EA or type III EA (e.g., with prematurity) underwent primary anastomosis associated with a systematic gastrostomy depending on the local team’s experience. The weight/age Z-score was 0.1 (range, −5.1 to 5.5; ±2.0) at 6 months and −0.8 (range, −5.5 to 3.7; ±1.2) at 12 months. Fifteen percent of patients were undernourished at the ages of 6 and 12 months, whereas 37% presented with respiratory symptoms and 15% had dysphagia at the last follow-up.

Comparative analyses of the children who presented with at least one complication (n = 91) compared with those with no complications (n = 180) are summarized in Table 3. Significant independent factors that were associated with a medical complication during the first-year follow-up were anastomotic esophageal tension (56% vs 44%; OR, 3.0 [1.6–5.6] p = .0009), presence of a gastrostomy (71% vs 29%; OR, 4.8 [2.1–11.1] p = .0002); exclusive oral feeding at discharge was associated with a decrease risk of complication (74% vs 26%; OR, 4.5 [1.5–14.2] p = .007). Factors such as prematurity, low birth weight, pure EA, and associated malformations were not significantly associated with complications.

**Table 3:** **Comparison between children presenting with and without complications during the first year of life**

	**Complications (n = 91)**	**No complications (n = 180)**	**p**	**Multivariate analysis**
**Sex (%)**	Male (31)	Male (69)	.8	NS
	Female (29)	Female (71)		
**Term (weeks)**	36.5 ± 3.3	37.6 ± 2.9	.005	NS
**Birth weight (g)**	2419 ± 710	2672 ± 677	.003	NS
**Associated malformations (%)**	57/141 (40)	84/141 (60)	.01	NS
**EA type (%)**	LADD I (64)	LADD I (36)	.01	NS
	LADD III (29)	LADD III (71)		
**Defect length**	1.6 ± 4.8	1.5 ± 1.2	.0002	NS
**Primary anastomosis (%)**	70/235 (30)	165/235 (70)	.003	NS
**Delayed primary anastomosis (%)**	22/32 (69)	10/32 (31)	1.4 10^−5^	NS
**Anastomotic tension (%)**	48/86 (56)	38/86 (44)	1.2 10^−7^	**OR, 3.0 [1.6–5.6] p = .0009**
**Difficult anastomosis (%)**	16/28 (57)	12/28 (43)	.01	NS
**Gastrostomy (%)**	40/56 (71)	16/56 (29)	4.9 10^−11^	**OR, 4.8 [2.1–11.1] p = .0002**
**Complementary enteral nutrition at discharge (%)**	28/40 (70)	12/40 (30)	2.9 10^−7^	**NS**
**Exclusive oral feeding at discharge (%)**	57/219 (26)	162/219 (74)	3.1 10^−6^	**OR, 4.5 [1.5-14.2] p = .007**

NS, not significant.

OR, odds-ratio.

## Discussion

The survival rate of EA in developed countries reached a plateau in the 1980s and seems to be currently stable at around 95% [2,3]. Major cardiac malformations and low birth weight (<1500 g) are two predictors identified by Spitz using the modification of Waterston’s risk-group classification, and these risk factors are consistent with those reported in the French National Register [4,7]. Death occurred during the first days of life in most of those who died (Table 2).

Our data from a population-based register show that more than one-third of patients operated on for EA had medical complications. Comparisons with the literature are difficult because the definition of complications differs between studies. For example, in a retrospective monocentric series, Castilloux et al. reported that more than half of their patients had a complicated evolution, but they considered severe gastroesophageal reflux confirmed at biopsy or severe chronic respiratory disease as complicated issues [8]. We decided to consider only reliable and objective complications to homogenize the results because the register involves 38 different centers and investigators. This showed clearly that despite recent advances in neonatal care and surgical techniques, morbidity remains high within the first year of life of EA patients.

Our study also showed that these patients required frequent rehospitalization during the first year compared with a previous study that reported a 67% hospitalization rate [9]. As expected, digestive and respiratory problems were the main causes of hospitalization [10]. Parents should be informed about this issue.

Complications directly related to esophageal anastomosis remain frequent, despite recent surgical progress [2,8,11-14]. Similarly, gastroesophageal reflux remains a major problem, as 10–20% of patients needed antireflux surgery within the first year of life [2,13,14].

Respiratory complications are also a major concern in children with EA and represent an important factor related to morbidity; respiratory disease at 1 year of age has been reported in 9% of patients [8]. These complications are often underestimated, and a systematic first-year survey found respiratory symptoms in 37% of our patients.

Nutritional status is rarely reported in EA populations, and data are lacking particularly in the first year of life. For example, Chetcuti et al. reported malnutrition in 13% of their patients with EA aged 1–37 years, of whom two-thirds were younger than 5 years [9]. Other authors have reported a growth delay in nearly one-third of these children at the age of 5 years [13]. All reported data apply only to older children with EA. Fifteen percent of our patients were undernourished at 6 and 12 months of life, which indicated that malnutrition did not decrease between 6 and 12 months of age despite earlier nutritional support.

One of the main results of our study was the identification of three independent predictors of complications. One neonatal factor was linked to the malformation and surgical procedure (esophageal anastomotic tension), and two nutritional factors were significantly related to the occurrence of complications. Anastomotic tension may favor gastroesophageal reflux and esophageal stenosis, whereas gastrostomy and enteral nutrition (naso-gastric tube) are often necessary in more difficult cases with esophageal dysmotility or anastomotic stenosis resulting in delayed food intake or malnutrition. Although anastomotic tension could not be evaluated using an objective methodology, we believe that the association with complications is valuable because this information was recorded prospectively without knowledge of the outcomes. Pure EA, prematurity, and associated malformations, which were associated with complications in previous studies, were not significant in the present study; however, our study might have been underpowered regarding these factors.

The purpose of identifying the early predictors of morbidity/mortality is to identify patients who require more intensive follow-up programs [8]. Because the survival rate has improved, these children with EA will gradually enter adulthood with significant esophageal morbidity [15]. Most of the teams that currently treat children with EA abandoned the original idea of “complete healing” once surgical anastomosis has been performed, and it is now suggested that the crucial role of multidisciplinary specialist clinics should be the long-term follow-up of these patients [13].

To our knowledge, this was the first prospective national registry to focus specifically on EA with a national dimension. One of the key points of this type of registry is its exhaustiveness, with few patients lost to follow-up and a large number of patients included in the analyses, which resulted in high statistical power. Some of the variables studied can be center dependent. However, the multicentric characteristic of our registry limits this bias and reflects the reality at a population level because the registry was not limited to selected populations from tertiary centers.

The results obtained from our population-based registry are important for health-care providers who are involved in prenatal and neonatal care because they may help to inform, and to provide better counseling to the parents of a fetus or newborn affected by EA.

## Conclusions

In conclusion, although mortality was low, digestive and respiratory morbidities were frequent in the first year after EA repair and often required rehospitalization. This situation is associated with high health costs and has social and psychological effects on the relatives of these children. Information provided to the parents should be adapted to highlight these risks. EA follow-up is necessary, especially during the first year of life. High-risk groups of patients were identified.

## Availability of supporting data

Supporting data are not available in a publicly-accessible data repository.

## Additional file

Additional file 1:
**Esophageal atresia French national registry: 1-year follow-up questionnaire.**
