# Supplementary material for: Results from the French National Esophageal Atresia register: one-year outcome
Source: Orphanet J Rare Dis. 2014 Dec 11;9:206. doi: 10.1186/s13023-014-0206-5 (PMC4265341; doi:10.1186/s13023-014-0206-5)
Supplement: Additional file 1: — Esophageal atresia French national registry: 1-year follow-up questionnaire. [file 13023_2014_206_MOESM1_ESM.doc]

**Center N°:**  **Patient N°: Name**  - () **First name -** ()

Deceased:  Yes  No Lost to follow-up:  Yes  No

Date of birth

Date of diagnosis

Date of death

***Complications ≤ 12 months***

Yes  No

Anastomotic leaks  Yes  No

Anastomotic stenosis  Yes  No

Esophageal dilation  Yes  No

Recurrent tracheo-esophageal fistula  Yes  No

Age at first dilation (days) Total number of dilations

***Follow-up ≤ 12 months***

Re-hospitalization(s)  Yes  No Number of hospitalizations

***Cause of hospitalization***

Hospital stay (days)

Main cause of hospitalization Respiratory  Yes  No

Digestive  Yes  No

***2nd intervention ≤ 12 months***

Anti-reflux surgery  Yes  No Age (days)

Aortopexy  Yes  No Age (days)

***Evaluation at 6 and 12 months***

**6 months** Weight (g) Height (cm)

**12 months** Weight (g) Height (cm)

Respiratory symptoms at 12 months  Yes  No  Unknown

Dysphagia at 12 months  Yes  No  Unknown

Date

Signature

|  |
| --- |
